# Compression of the radial nerve at the elbow by a ganglion: two case reports

**DOI:** 10.4076/1752-1947-3-7258

**Published:** 2009-06-05

**Authors:** I-Ming Jou, Hung-Nan Wang, Ping-Hui Wang, Ing-Sing Yong, Wei-Ren Su

**Affiliations:** 1 Department of Orthopaedic Surgery, National Cheng Kung University Hospital, Tainan 704, Taiwan; 2 Department of Nursing, Min-Hwei College of Health Care Management, Tainan County 736, Taiwan; 3 Department of Orthopaedic Surgery, Chi-Mei Medical Center, Tainan County 710, Taiwan; 4 Department of Orthopaedic Surgery, SinLau Hospital, Tainan, Taiwan

## Abstract

**Introduction:**

Radial nerve compression by a ganglion in the radial tunnel is not common. Compressive neuropathies of the radial nerve in the radial tunnel can occur anywhere along the course of the nerve and may lead to various clinical manifestations, depending on which branch is involved. We present two unusual cases of ganglions located in the radial tunnel and requiring surgical excision.

**Case presentation:**

A 31-year-old woman complained of difficulty in fully extending her fingers at the metacarpophalangeal joint for 2 weeks. Before her first visit, she had noticed a swelling and pain in her right elbow over the anterolateral forearm. The extension muscle power of the metacarpophalangeal joints at the fingers and the interphalangeal joint at the thumb had decreased. Sonography and magnetic resonance imaging of the elbow revealed a cystic lesion located at the area of the arcade of Frohse. A thin-walled ovoid cyst was found against the posterior interosseous nerve during surgical excision. Pathological examination was compatible with a ganglion cyst. The second case involved a 36-year-old woman complaining of numbness over the radial aspect of her hand and wrist, but without swelling or tumor in this area. The patient had slightly decreased sensitivity in the distribution of the sensory branch of the radial nerve. There was no muscle weakness on extension of the fingers and wrist. Surgical exposure defined a ganglion cyst in the shoulder of the division of the radial nerve into its superficial sensory and posterior interosseous components. There has been no disease recurrence after following both patients for 2 years.

**Conclusion:**

Compression of nerves by extraneural soft tissue tumors of the extremities should be considered when a patient presents with progressive weakness or sensory changes in an extremity. Surgical excision should be promptly performed to ensure optimal recovery from the nerve palsy.

## Introduction

Compressive neuropathies are important and widespread debilitating clinical problems. The two most common compressive peripheral nerve disorders in the upper limb are carpal tunnel syndrome and cubital tunnel syndrome, however, radial tunnel syndrome occurs less frequently [1]. The radial tunnel is defined as the potential space created by structures surrounding the radial nerve as well as its posterior interosseous nerve and its superficial sensory branch as they travel through the proximal forearm from the radiocapitellar joint past the proximal edge of the supinator muscle [1,2]. Compressive neuropathies of the radial nerve in the radial tunnel can occur anywhere along the course of the nerve and may lead to various clinical manifestations, depending on which branch is involved [3,1]. Radial nerve compression by a ganglion in the radial tunnel is not common [4]. The clinical features of our two cases of radial nerve compression syndrome due to a ganglion are reported, and the anatomical characteristics of two possible compression sites in the radial tunnel are discussed.

## Case presentation

### Patient 1

**Figure 1:**
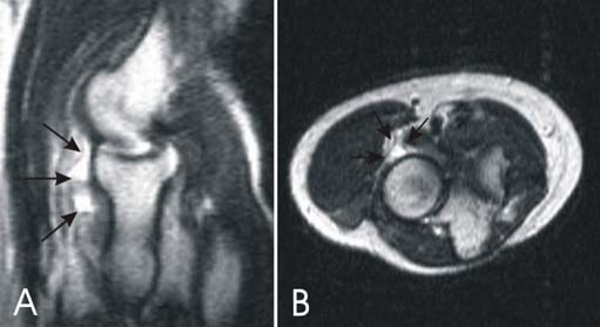
****(A)** Sagittal T2W image at the level of the radiocapitellar articulation reveals the extent and lobulated morphology of the cystic lesion (arrows), which is located directly anterior to the anterior aspect of the radial head**. **(B)** Axial, T2W image at the level of the radial head demonstrates a lobulated cystic lesion (arrows) in the expected area of the posterior interosseous nerve.

A 31-year-old woman complained of difficulty in fully extending her fingers at the metacarpophalangeal joint for 2 weeks. Before her first visit, she had noticed swelling and pain in her right elbow over the anterolateral forearm. Examination revealed a tender swelling in the anterolateral region of the antecubital fossa but no clinical evidence of a mass. The extension muscle power of the metacarpophalangeal joints at her fingers and the interphalangeal joint at her thumb had decreased. The patient had full strength with resisted wrist extension and resisted supination. The radial nerve sensibility was normal. An electromyogram and nerve conduction study showed early partial neuropathy of the posterior interosseous nerve. Sonography revealed a mass continuous with the posterior interosseous nerve immediately distal to the radiocapitellar joint. Magnetic resonance imaging (MRI) of the elbow revealed a cystic lesion located at the area of the arcade of Frohse, which was attached to the anterior capsule of the radiocapitellar joint (Figure 1A, B). Due to progression of symptoms and limitation in daily activities of the patient, surgical excision was performed. The cyst and radial nerve were explored through an anterolateral incision. A thin-walled ovoid cyst, 2 × 1.5 × 0.8 cm in size, was found against the posterior interosseous nerve just proximal to the arcade of Frohse. The cyst was identified as a ganglion and excised completely. The diagnosis of a ganglion was confirmed histologically. Two years postoperatively, there has been no evidence of recurrence and the patient has returned to her former activities.

### Patient 2

**Figure 2:**
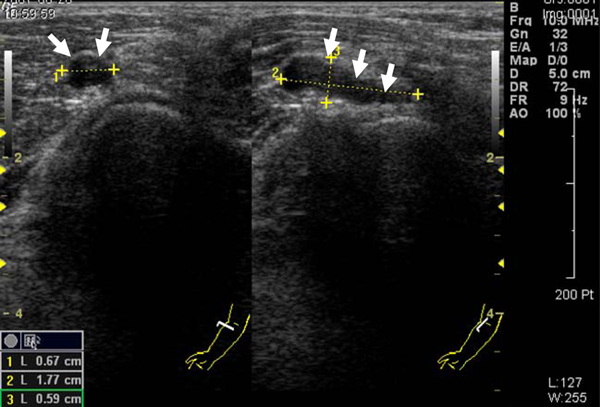
**Sonogram showing a cystic lesion reveals a 0**.6 × 0.7 × 1.8 cm hypoechoic mass that is just anterolateral to the radial head. The cystic lesion is likely causing a mass effect against the sensory branch of the radial nerve.

A 36-year-old, right-handed woman complained of numbness over the radial aspect of her hand and wrist. She complained of pain in the lateral aspect of her elbow but did not notice swelling or tumor in this area. On physical examination, a mass could not be definitely detected by palpation. The patient had slightly decreased sensitivity in the distribution of the sensory branch of the radial nerve. There was no muscle weakness on extension of her fingers and wrist. Electrodiagnostic studies were consistent with the diagnosis of neuropathy of the sensory branch of the radial nerve. Ultrasonography and MRI each demonstrated a well-defined mass just anterior to the radiocapitellar joint (Figure 2). Surgical exposure through the anterolateral elbow defined a ganglion cyst in the shoulder of the division of the radial nerve into its superficial sensory and posterior interosseous components. The ganglion was excised with its base. Sensory function was completely restored within 2 months after surgery.

## Discussion

Radial nerve entrapment in the radial tunnel is uncommon in peripheral nerve compressive neuropathies. There are three different types of palsy in the radial tunnel syndrome: posterior interosseous nerve palsy, neuropathy of the sensory branch of the radial nerve, and neuropathy of both nerves [1,3]. The posterior interosseous nerve is most vulnerable to compression just beyond its origin as it passes beneath the arcade of Frohse at the proximal edge of the supinator in the radial tunnel [3]. Compression of the posterior interosseous nerve alone may manifest as motor weakness in the distribution of the posterior interosseous nerve, resulting in inability to extend the metacarpophalangeal joints of the finger and thumb, as well as weakness in extension of the thumb at the interphalangeal joint, also called "finger drop". Usually there is not complete wrist drop, because the extensor carpi radialis longus and brevis are supplied by the radial nerve proximal to its terminal branch. Compression of the superficial sensory branch alone may present as pain and decreased sensation along the cutaneous area on the radial side of the dorsum of the hand [5]. When a patient presents with compression neuropathy of the radial nerve below the elbow, differential diagnosis of the cause of the palsy and further determination of the location of the entrapment in the radial tunnel are important.

**Figure 3:**
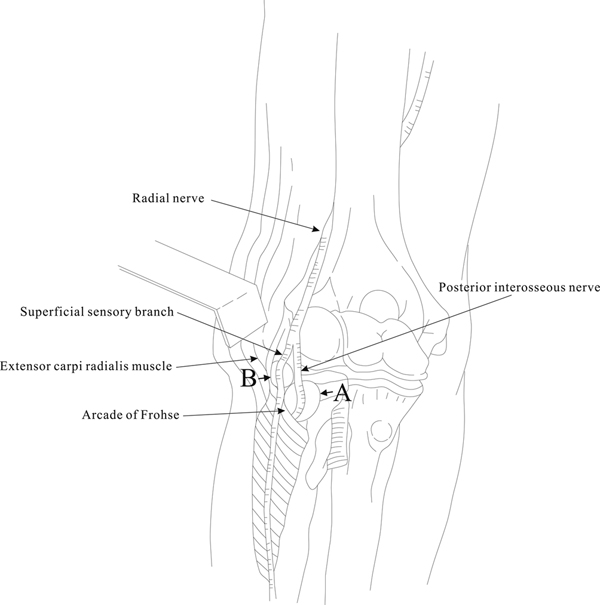
**Diagrammatic view of the radial tunnel showing compression of the ganglion on various locations of the branches of the radial nerve**. Arrow A indicates the compression present in patient 1 and arrow B indicates the compression present in patient 2.

Our first case experienced lateral forearm pain that is initially often difficult to distinguish from lateral epicondylitis, synovitis of the radiocapitellar joint, and a muscle tear of the extensor carpi radialis brevis [2]. However, due to the progressive weakness of finger extension with sparing of the wrist extension function and the sensory of the radial nerve, posterior interosseous nerve palsy alone was thought to be the underlying disease. Our second case was diagnosed as compressive neuropathy of the superficial branch of the radial nerve, which can give rise to pain within the distribution of the superficial radial nerve, and could mimic de Quervain's tendovaginitis stenosans. Our operative findings in each case revealed that a ganglion arose from the anterior capsule of the elbow, to push up the deep or superficial sensory branches of the radial nerve, compressing the deep branch against the arcade of Frohse and the superficial branch against the extensor carpi radialis muscle (Figure 3). Yamazaki *et al.* reported 14 patients presenting with posterior interosseous nerve (PIN) paralysis due to a ganglion at the elbow, located proximal to the proximal edge of the supinator muscle in 13 cases and distal in one [6]. These facts suggest that there is a possibility that palsy of either the posterior interosseous nerve or the radial sensory branch may occur anywhere along the radial tunnel whenever a ganglion is present.

Compression of the posterior interosseous nerve by a ganglion was first reported by Bowen in 1966 [7]. He recorded a ganglion developed from post-traumatic osteoarthritic elbow secondary to an old intercondylar fracture of the humerus. These cysts are most likely caused by repetitive use or by inflammatory or traumatic conditions, and result primarily from myxoid degeneration. They are associated with increasing liquefaction of collagen fibers surrounded by densifying collagen bundles, which form a delimiting capsule. Other tumors can result in a space-occupying lesion which compresses the nerve. The most common tumors causing symptoms are rheumatic synovial cysts, lipomas, fibromas and pseudoneuromas [7]-[9]. Usually, these tumors cause paralysis with an insidious onset.

When a ganglion is not detected by palpation in cases with palsy of the posterior interosseous nerve or sensory branch, differential diagnosis of the cause of the palsy may be difficult. Compression of nerves by extraneural soft-tissue tumors of the extremities, although not common, should be considered when a patient presents with progressive weakness or sensory changes in an extremity. This is true whether or not a soft-tissue mass is found during examination, since an occult soft-tissue tumor was found in approximately one-third of patients at the time of operation in a report by Barber [10].

Ultrasound and MRI have been used to detect space-occupying lesions causing nerve compression [4]. Ultrasound allows a detailed assessment of peripheral nerve continuity with a mass, which indicates an intrinsic nerve abnormality rather than an adjacent extrinsic mass. Ultrasound has also been used to determine whether a lesion is cyst or solid. Recent advances in MRI have increased the ability to localize the region of the nerve compression, and assess the tumor's relationship to nearby neurovascular structures, enhancing pre-operative planning for a precise surgical excision.

Standard surgical management for persistent neuropathy, refractory to non-surgical treatment, is open decompression of the radial nerve. This can be done through a variety of anterior or lateral approaches. The approach includes addressing all of the potential sites of compression in addition to excising the mass lesions described previously which may cause compression of the radial nerve.

## Conclusion

Radial nerve compression by a ganglion in the radial tunnel is an uncommon condition. We report two cases of posterior interosseous nerve and superficial sensory branch compression by a ganglion cyst. The value of this case report to the practicing physician is that it sheds light on the importance of familiarity with the possible presentation, anatomic location and differential diagnosis to facilitate corrective diagnostic approaches and timely management.

## Consent

Written informed consent was obtained from both patients for publication of this case report and any accompanying images. A copy of the written consent is available for review by the Editor-in-Chief of this journal.

## Competing interest

The authors declare that they have no competing interests.

## Authors' contributions

IMJ participated in the surgical interventions, contributed to the study concept and design and drafted the manuscript. HNW participated in the medical interventions, took the photographs and undertook the literature review. ISY was involved in reviewing the histological section of the case and proofreading of the manuscript. WRS participated in the surgical interventions and helped draft the final version of this manuscript to be published. All authors read and approved the final manuscript.
